# Supplementary material for: Electrically driven amplification of terahertz acoustic waves in graphene
Source: Nat Commun. 2024 Mar 21;15:2550. doi: 10.1038/s41467-024-46819-2 (PMC10957956; doi:10.1038/s41467-024-46819-2)
Supplement: Supplementary file 1 — Supplementary Information [file 41467_2024_46819_MOESM1_ESM.pdf]

Supplementary Information

Electrically driven amplification of terahertz acoustic waves in graphene

Aaron H. Barajas-Aguilar, Jasen Zion, Ian Sequeira, Andrew Z. Barabas, Takashi Taniguchi, Kenji Watanabe, Eric B. Barrett, Thomas Scaffidi, and Javier D. Sanchez-Yamagishi.

Contents

Supplementary Note 1: Additional data on device A. 2
1.1 Distance dependence of differential resistivity 2
1.2 Exponential resistivity growth with distance and total resistance calculation. 2
1.3 Carrier density dependence of the differential resistivity for different pairs of contacts 3
1.4 Temperature dependence for contacts 5-6. 4
1.5 Plot of the emitted phonon frequencies 5
1.6 Two probe measurements. 6
Supplementary Note 2: Phonon amplification in Device B, second non-aligned device. 6
Supplementary Note 3: Device C, graphene/hBN aligned device. 9
Supplementary Note 4: Device D, disordered device. 10
Supplementary Note 5: Theory calculations. 11
5.1 Phonon amplification rate 12
5.2 Phonon distribution 13
5.3 Resistivity 14

Supplementary Note 1: Additional data on device A.

1.1 Distance dependence of differential resistivity

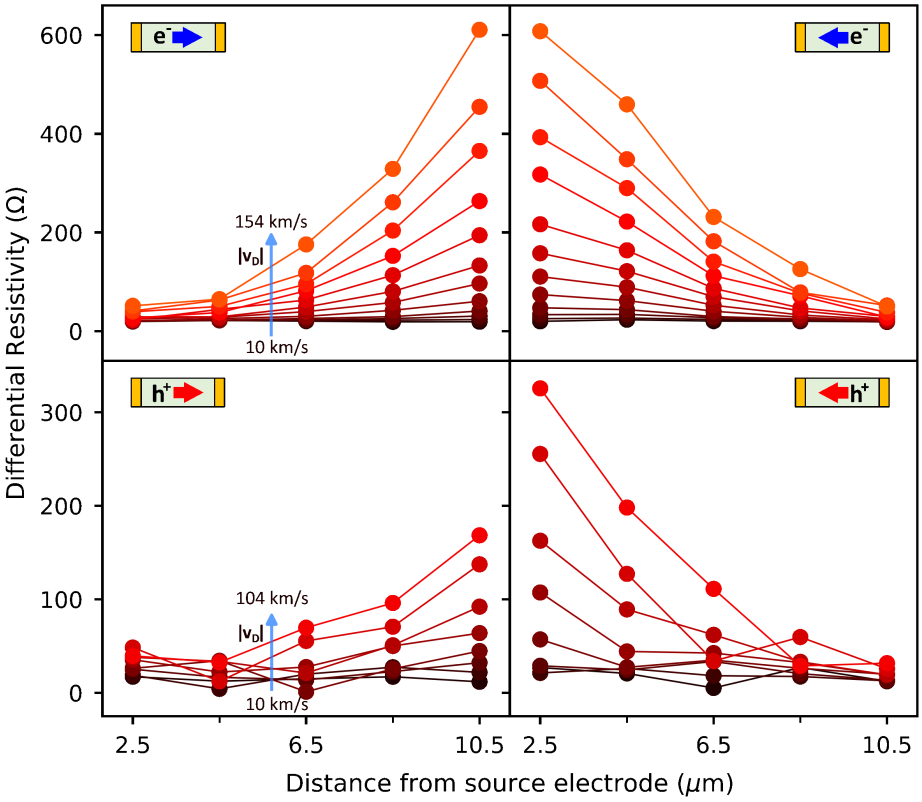

Supplementary Figure 1. Distance dependence of differential resistivity for device A at carrier densities of 1.4×10^{12} cm^{-2} (top panel) and n=-1.4×10^{12} cm^{-2} (bottom panel). In the top panels the maximum current density (j=0.34 mA/μm) corresponds to a v_D=154 km/s and a 12x increase of the differential resistivity in 8 μm can be appreciated. For the bottom panels the maximum v_D value is 104 km/s.

1.2 Exponential resistivity growth with distance and total resistance calculation.

The resistivity vs. distance curve for v_D=154 km/s for right-moving electrons (top-left panel of Fig.1d) was fitted by an exponential function of the type $r=m+ae^{(b*x)}$ (Supplementary Fig. 2). The parameters a, b and m were fitted using a nonlinear least squares method. From this fit, we calculate the total resistance of the graphene channel to be 481 Ω at v_D = 154 km/s, where 81% of that resistance comes from the last 6 μm of the 13 μm long device.

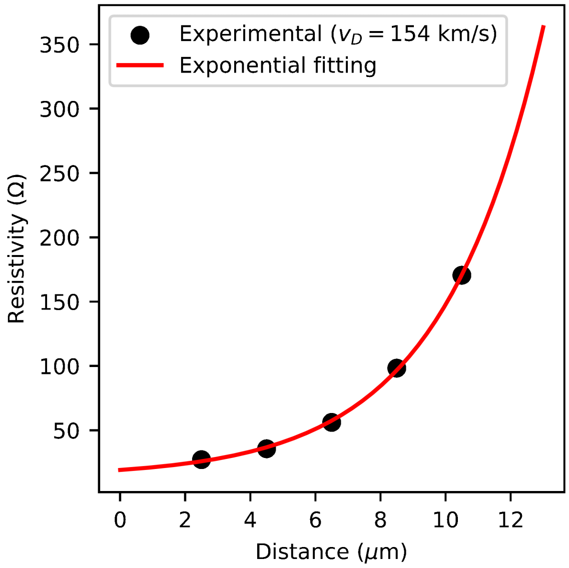

Supplementary Figure 2. Exponential fitting of the resistivity vs. distance curves. The fitted parameters for the curve are: **v_D=154 km/s**, a=5.49±1.13 Ω, b=0.32±0.02 μm^{-1}, m=13.68±3.33 Ω.

1.3 Carrier density dependence of the differential resistivity for different pairs of contacts

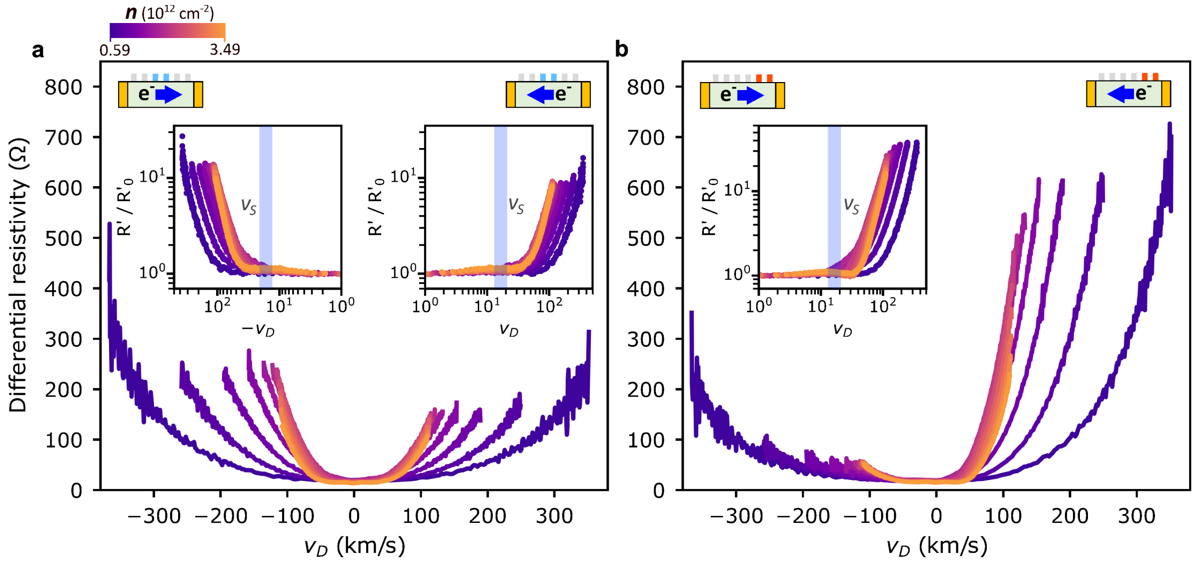

Supplementary Figure 3. Resistivity vs. drift velocity at different carrier densities for contacts 3-4 (a) and 5-6 (b). Panel a and b show the differential resistivity vs. v_D at different carrier concentrations for contacts 3-4 and 5-6 respectively of device A. For both pairs of contacts, a sharp transition between ohmic and non-ohmic behavior can be observed in the logarithmic insets (differential resistivity normalized to the value at v_D=0). The shadowed regions indicate drift velocities between 13 and 21 km/s, corresponding to the speed of sound for TA and LA phonons respectively. The device schemes indicate the carrier flow direction, the type of carriers and the contacts being measured in each case (colored contacts).

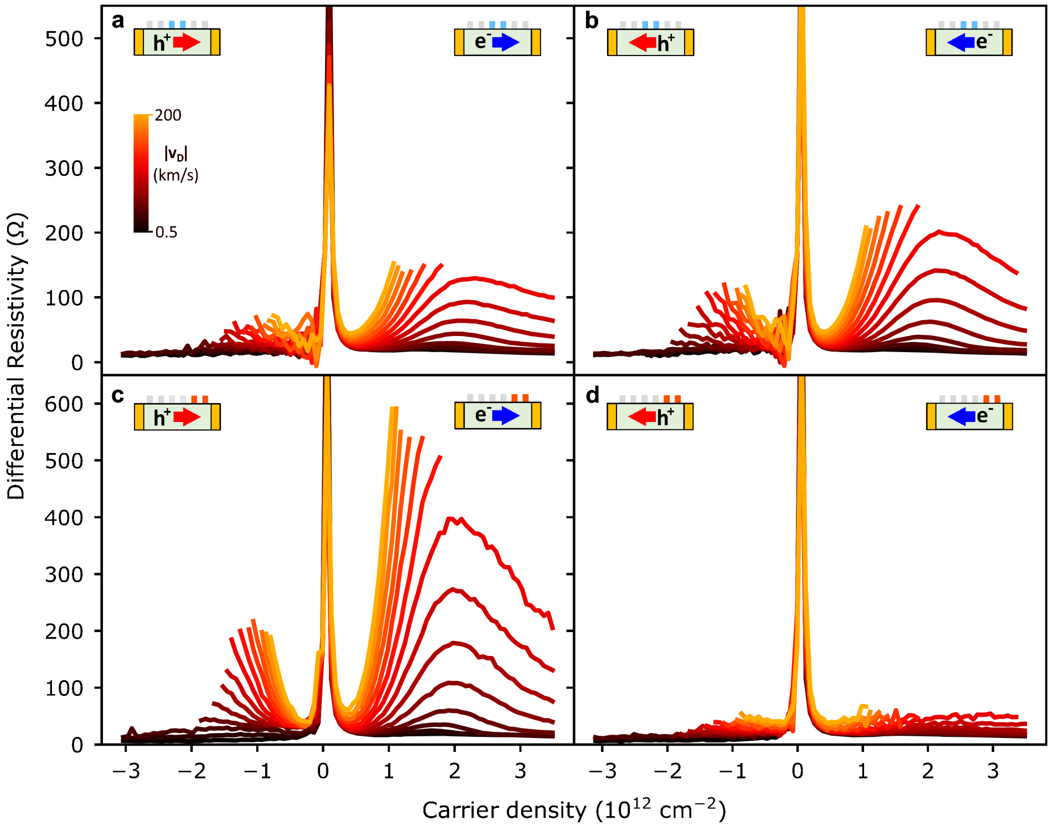

Supplementary Figure 4. Resistivity vs. Carrier density for contacts 3-4 and 5-6. The top panel (a, b) shows the differential resistivity vs. carrier concentration for contacts 3-4 in both directions of the carrier flow. Since these contacts are located at the center of the device, phonon amplification is observed for both directions, however, the resistivity growth is not as large as in contacts 1-2 or 5-6. The bottom panel shows the case for contacts 5-6. As can be observed, just like in the case of contacts 1-2, there is large growth of the differential resistivity when the carriers flow downstream (c). On the other hand, the resistivity growth is barely noticeable if the direction of the current is reversed (d). The device cartoons indicate the carrier flow direction, the type of carriers and the contacts being measured in each case (colored contacts). Note that data were taken within a maximum source-drain voltage of ±0.6 V, hence the curves appear with different ranges when plotted versus current, v_D, or for constant values of v_D.

1.4 Temperature dependence for contacts 5-6.

Supplementary Figure 5 shows the temperature dependence for contacts 5-6 at a carrier density of 1.4×10^{12} cm^{-2}. The differential resistivity vs. v_D at different temperatures (a) and resistivity vs. temperature curves (b) are similar to those of contacts 1-2, except that in this case the resistivity growth occurs for right-moving carriers. In Supplementary Figure 5a, the asymmetry of the differential resistivity vs. v_D curve at 280 K is still evident, just as observed for contacts 1-2 (Fig. 3a).

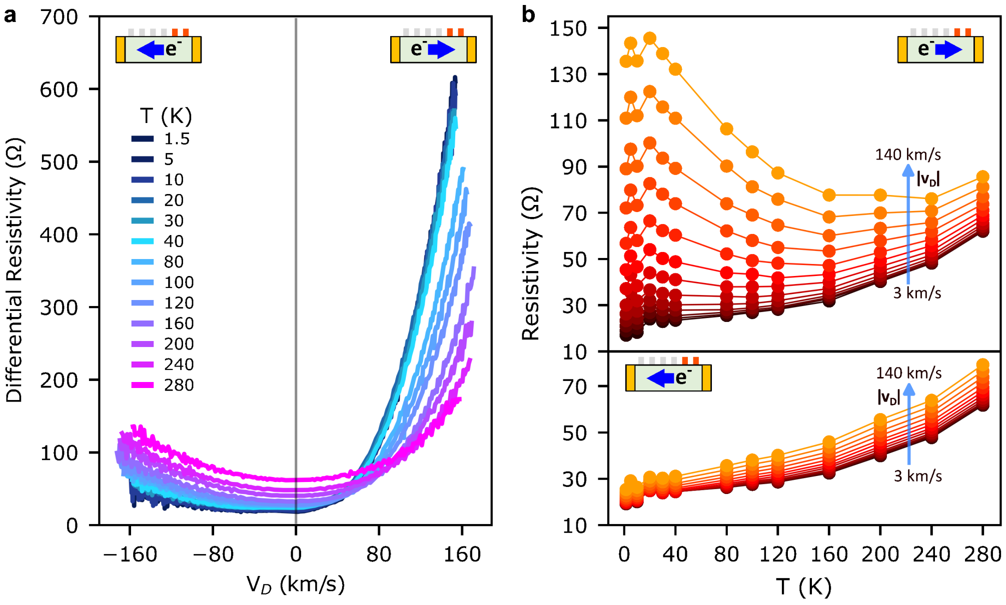

Supplementary Figure 5. Temperature dependence at *n*=1.4×10^{12} cm^{-2} for contacts 5-6. a) Differential resistivity vs. v_D at different temperatures. b) Resistivity vs. *T* at different v_D values.

1.5 Plot of the emitted phonon frequencies

As mentioned in the main text the energy of the emitted phonons can be estimated as E=ħv_S*2k_F, where $k_F=\sqrt{\pi n}$. Supplementary Figure 6 shows the curves for the longitudinal and transversal phonons. The minimum frequency reported in the main text corresponds to that of the transversal phonons at 0.4x10^{12} cm^{-2}, the lowest carrier density where we measure phonon amplification. The maximum frequency corresponds to that of the longitudinal phonons at the higher carrier density reached in the measurements of device A (3.5x10^{12} cm^{-2}).

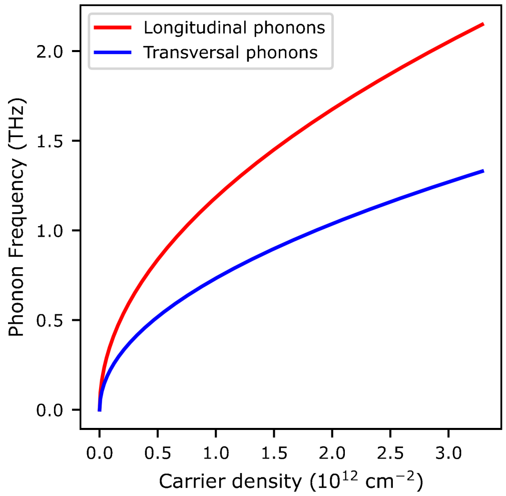

Supplementary Figure 6. Frequency of emitted longitudinal and transversal acoustic phonons vs. carrier density.

1.6 Two probe measurements.

When the applied source-drain voltage on a device is comparable to the gate voltage, self-gating effects can induce a significant variation of the carrier density along the device, producing a distance dependent resistivity profile. In our case, the maximum applied SD voltage is 0.6 V, which is small compared to the gate voltages applied when phonon amplification is clearly observed, this is, from ~6 to 50 V, corresponding to carrier densities from $0.6x10^{12}$ to $3.5x10^{12}$ cm^{-2} (Fig.2d). Supplementary Figure 7 shows the applied source-drain bias vs. current and the two-probe differential resistivity vs. current at different carrier densities.

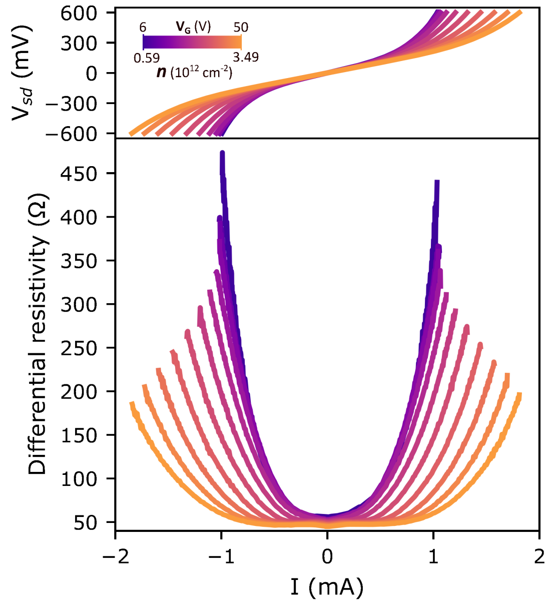

Supplementary Figure 7. Two probe measurements for device A. Applied source drain voltage vs. current (top) and two probe measurements of the differential resistivity vs. current (bottom) at different carrier densities. The curves appear symmetric, as the spatial growth of the resistance is hidden in the total two-probe resistance of the device.

Supplementary Note 2: Phonon amplification in Device B, second non-aligned device.

Device B is the second unaligned device presented in this work. Like device A, it also exhibits clear signs of phonon amplification (Supplementary Figs. 8, 9 and 10). First, we determine that the graphene is unaligned with the hBN from resistivity versus carrier density measurements which show a typical graphene Dirac peak (Supplementary Fig. 8b). When biased, we observe the same qualitative features measured in Device A presented in the main text figures:

(1) The *V-I* curves are highly asymmetric due to the local resistivity growing in the carrier flow direction when the drift velocity exceeds the sound velocity (Supplementary Fig. 8).

(2) The onset of non-ohmic behavior occurs with a sharp threshold only above the sound velocity (Supplementary Fig. 9b).

(3) The resistance growth occurs away from the Dirac point and increases with carrier density (Supplementary Fig. 9c). Note, unlike in Device A, we do not observe the resistance growth decreasing again at higher carrier densities.

(4) The asymmetric resistance growth is enhanced at low temperatures. The excess resistivity induced by biasing at low temperatures is larger than that induced by heating to room temperature (Supplementary Fig. 10).

A difference is that device B is clearly more disordered than device A, which we observe as a spatially varying resistivity across the device channel at zero bias (4 Ω for contacts 1-2 and 22 Ω for 4-5) that does not depend on the direction of the current. Therefore, Device B's resistance profile in Supplementary Figure 8d, includes the combined effects of both the phonon growth and the disorder; inverting carrier direction also flips the phonon growth profile, but has no effect on the disorder profile. The disorder profile is more evident at low $|v_D|$ values where the phonon amplification effects are not very strong, therefore, the resistivity increases towards the drain contact regardless of the direction of the current. For this reason, right-moving carriers show a larger resistance growth than left-moving carriers, consistent with both the phonon growth model and the pattern of disorder. By contrast, Device A has a uniform resistivity that varies by only 6% across the channel under zero bias conditions, and the right-moving and left-moving carriers have similar spatial profiles.

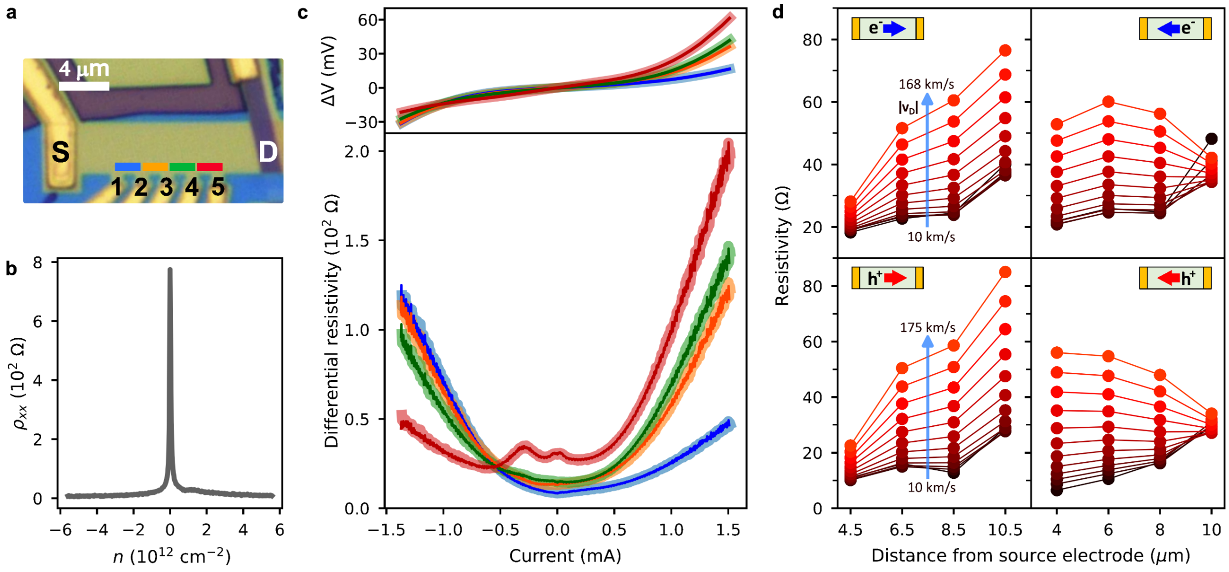

Supplementary Figure 8. Device B, *V-I* curves, and resistivity distance dependence. a) Optical image of device B with dimensions L=14.5 µm, w=4 µm and center to center distance between voltage tabs of 2 µm. The color code in this image is used to identify the line traces corresponding to each pair of contacts in panel c, S and D indicate the source and drain electrodes respectively. **b)** Dirac peak for device B, the absence of satellite peaks indicates that the graphene is not aligned with any of the hBN layers. **c)** Raw V-I curves (top) and differential resistivity vs. source-drain current (bottom) for all the pairs of contacts in device B at n=-1.4×10^{12} cm^{-2} (hole doped, Vg=-21 V). The lighter thick lines correspond to the forward current (from source to drain) while the solid thin lines are for the case of reverse current, where the source and drain cables are swapped. **d)** Length dependence of the differential resistivity at n=1.4×10^{12} cm^{-2} (top panel) and n=-1.4×10^{12} cm^{-2} (bottom panel). The device cartoons in each plot show the flow direction and type of carriers for each case. The green arrows indicate the minimum and maximum drift velocities for top and bottom panels. All measurements are performed at T= 2.8 K.

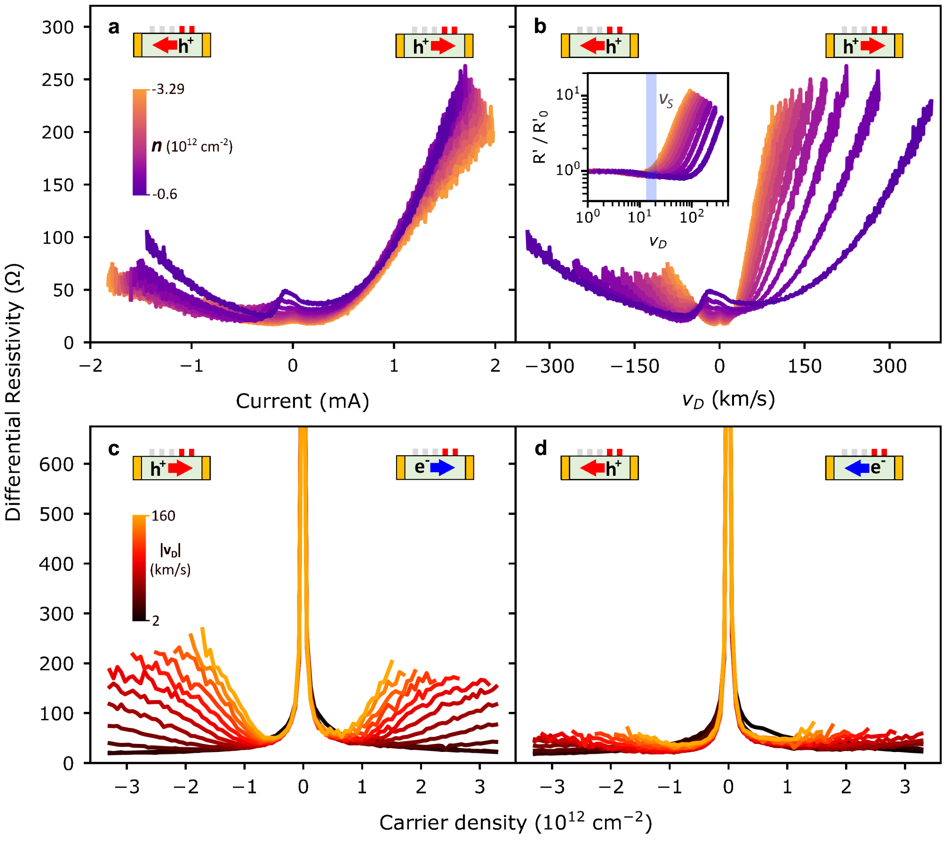

Supplementary Figure 9. Carrier density dependence for device B, contacts 4-5. Top: Differential resistivity as a function of **a)** current and **b)** drift velocity at different carrier densities, inset: normalized differential resistivity. Bottom: Differential resistivity vs. carrier density for **c)** downstream and **d)** upstream carrier flow. The device cartoons indicate the carrier flow direction, type of carriers and contacts being measured (colored contacts). Note that data were taken within a maximum source-drain voltage of ±1 V, hence the curves appear with different ranges when plotted versus current, v_D, or for constant values of v_D.

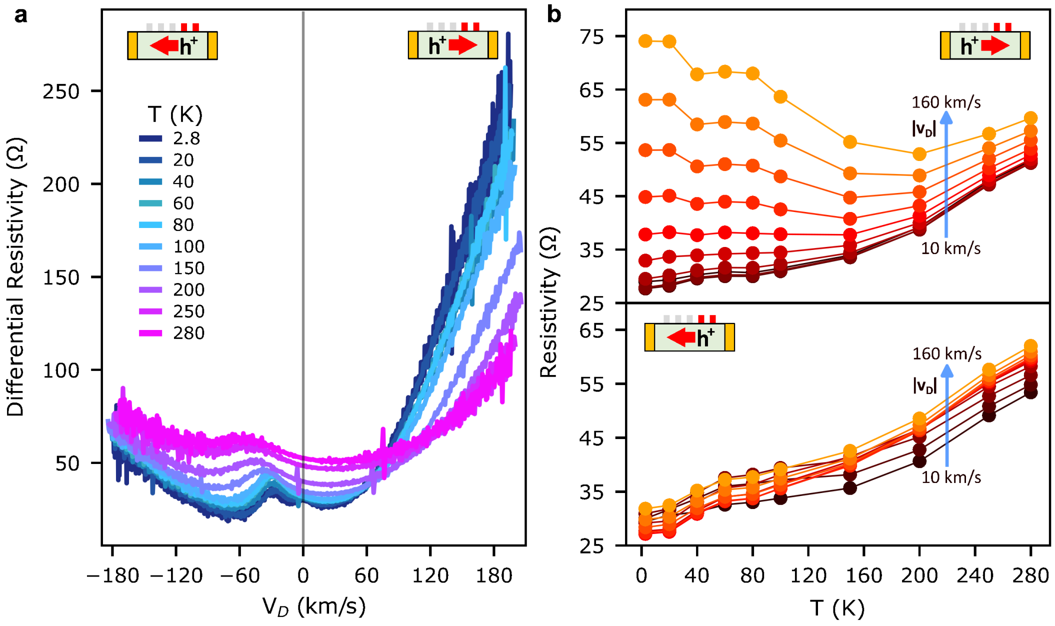

Supplementary Figure 10. Temperature dependence of device B at n=-1.4×10^{12} cm^{-2} for contacts 4-5. a) Differential resistivity vs. v_D curves at different temperatures. b) Resistivity vs. T at different v_D values.

Supplementary Note 3: Device C, graphene/hBN aligned device.

Supplementary Figure 11 shows the measurements taken for device C, which is fabricated identically to devices A & B, but has the graphene aligned to at least one of the hBN layers. Evidence for alignment comes from the resistivity vs. carrier density trace, which shows clear satellite resistance peaks and an overall higher resistivity of the device (Supplementary Fig. 11b). This indicates that at least one of the hBN layers is aligned at some low angle to the graphene layer, producing a 14 nm length scale moiré. This aligned device C shows extremely different transport behavior than the non-aligned devices A & B. The differential resistivity vs. current curves are symmetric, and do not show the characteristic asymmetry seen in non-aligned devices, that is the hallmark of phonon amplification (Supplementary Fig. 11c). Likewise, the spatial dependence of the differential resistivity does not depend on the direction of the current (Supplementary Fig. 11d), and likely arises from inherent sample inhomogeneity, such as twist angle variations. In the case of this aligned device, the nonlinearity of the V-I curve likely arises from Joule heating and Umklapp scattering[1]. In summary, there is no evidence of phonon amplification occurring in the aligned device C.

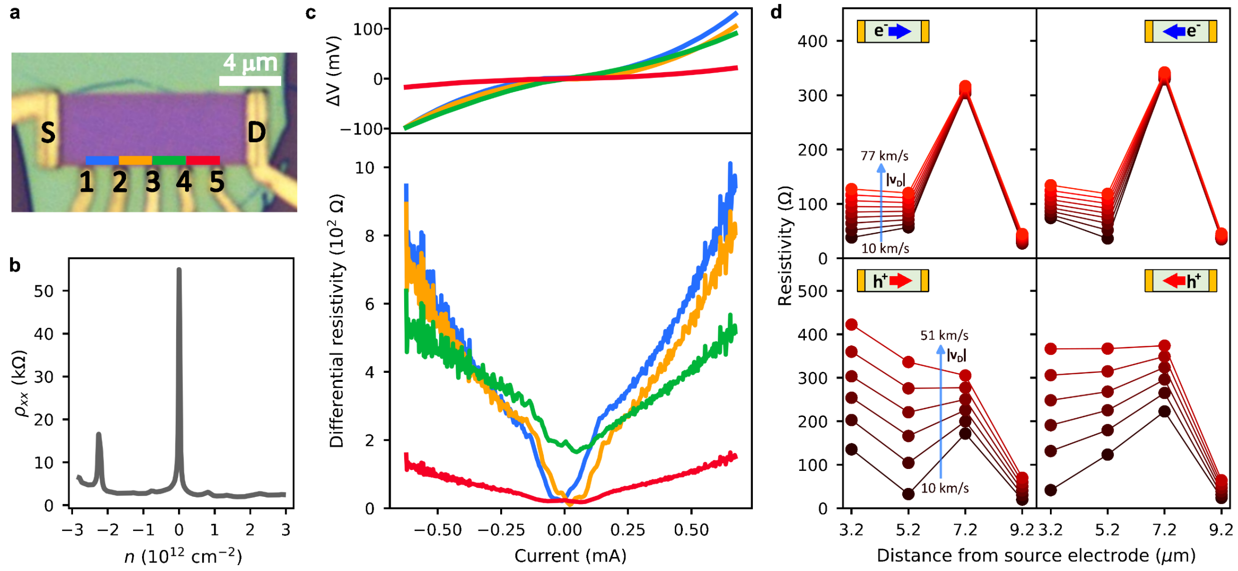

Supplementary Figure 11. Device C. a) Optical image of device C, the color code in this image is used to identify the line traces corresponding to each pair of contacts in panel c, S and D indicate the source and drain electrodes respectively. **B)** Dirac peak for device C, the satellite peaks indicate that the graphene is aligned with any of the hBN layers. **C)** Raw *V-I* curves (top) and differential resistivity vs. source-drain current (bottom) for all the pairs of contacts in device C at n=-1.4×10^{12} cm^{-2} (hole doped, V_G=-25 V). **d)** Length dependence of the differential resistivity at n=1.4x10^{12} cm^{-2} (top panel) and n=-1.4×10^{12} cm^{-2} (bottom panel). The device cartoons in each plot show the flow direction and type of carriers for each case. All measurements are performed at T = 1.5 K.

Supplementary Note 4: Device D, disordered device.

Device D is fabricated identically to A and B but exhibits 10x lower mobility (at n=1.4×10^{12} cm^{-2} and T=2.8 K), which varies across the device from 1.8 m^2/V*s to 2.78 m^2/V*s for contacts 1-2 and 3-4 respectively. The device shows substantial variation in the local resistance, as well as an optically non-uniform appearance which suggests the presence of impurities from the fabrication process. It does not exhibit any signs of graphene-hBN alignment. The V-I curves show only weak nonlinearities that are symmetric with current direction. The lack of resistance growth in this disordered sample supports the phonon amplification model, where sample disorder would be expected to scatter phonons and inhibit the amplification process.

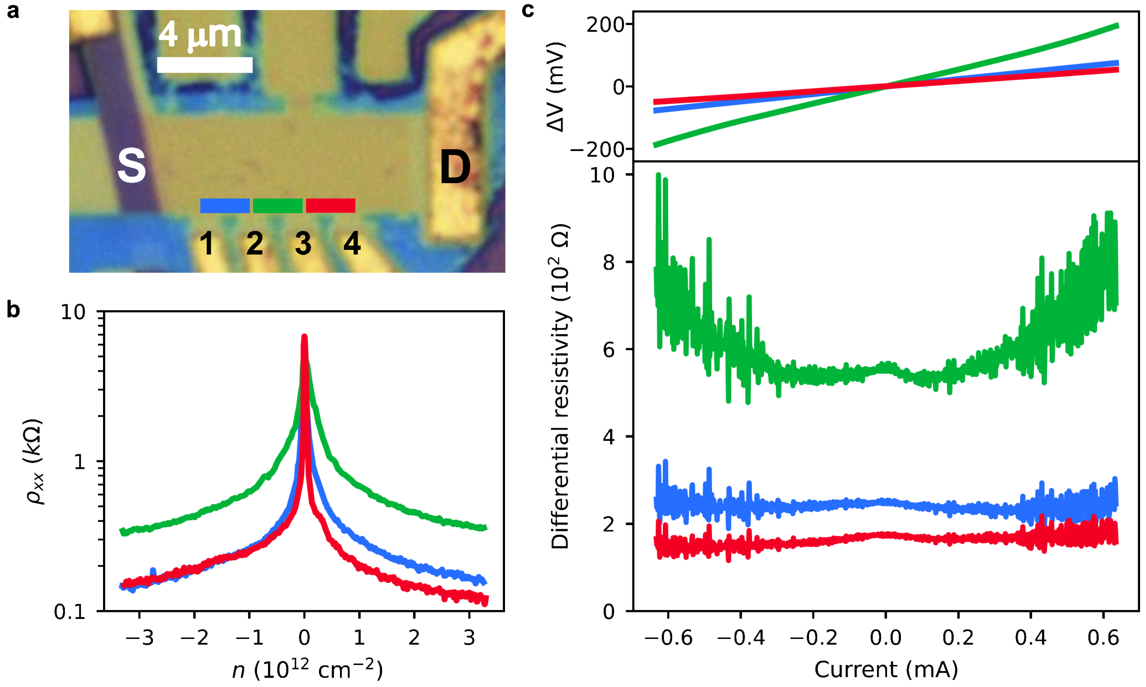

Supplementary Figure 12. Device D. a) Optical image of device D, the color code in this image is used to identify the line traces corresponding to each pair of contacts in panels b and c. **b)** Logarithmic scale Dirac peaks for the different pairs of contacts in device D. **c)** Raw V-I curves (top) and differential resistivity vs. source-drain current (bottom) for all the pairs of contacts in device D at n=1.4×10^{12} cm^{-2} (electron doped, V_G=21 V). All measurements are performed at T = 2.8 K.

Supplementary Note 5: Theory calculations.

Our calculation works in three steps. First, we assume a drifting Fermi–Dirac distribution with drift velocity v_d for the electrons in order to calculate the phonon amplification rate $\Gamma_{\mathbf{q}}^{\text{amp}}$ due to electron-phonon coupling. Second, we use $\Gamma_{\mathbf{q}}^{\text{amp}}$ to calculate the position-dependent out-of-equilibrium phonon distribution in the sample. Finally, we find the (position-dependent) electric field needed to sustain the assumed v_d based on the electronic Boltzmann equation in which the phonon distribution enters through the electron-phonon scattering integral. Our calculation is intended to reproduce the main physical features of the system with a minimum amount of details, and as such has limitations. The main one is our assumption of a drifting Fermi–Dirac distribution for the electrons, with a nominal temperature which is v_d-independent. In the actual system, we would expect that Joule heating would lead to increased electronic temperatures at high v_d, which could be considered in a refined calculation which treats electrons and phonons in a self-consistent way. Such a calculation is left for future work.

5.1 Phonon amplification rate

Phonons with a given wavevector $\mathbf{q}$ are amplified by the electron-phonon interaction with a characteristic amplification rate[2]:

$$\begin{aligned}\Gamma_{\mathbf{q}}^{\mathrm{amp}} &= \gamma_{\mathbf{q}}^{\mathrm{em}} - \gamma_{\mathbf{q}}^{\mathrm{abs}} - \tau_{\mathbf{q}}^{-1} \\ &= \left[\frac{2\pi}{\hbar} g_{\mathrm{s}} g_{\mathrm{v}} \sum_{\mathbf{k}} \sum_{\mathbf{k}'} \left|C_{\mathbf{k},\mathbf{k}',\mathbf{q}}\right|^2 \cdot [f_{\mathbf{k}}(1-f_{\mathbf{k}'}) - (1-f_{\mathbf{k}})f_{\mathbf{k}'}] \cdot \delta_{\mathbf{k}-\mathbf{k}',\mathbf{q}} \cdot \delta\left(E_{\mathbf{k}} - E_{\mathbf{k}'} - \hbar\omega_{\mathbf{q}}\right)\right] - \tau_{\mathbf{q}}^{-1},\end{aligned}$$

where:

$$\left|C_{\mathbf{k},\mathbf{k}',\mathbf{q}}\right|^2 = \frac{D^2\hbar|\mathbf{q}|}{2\rho A v_{\mathrm{s}}} \cos^2\left(\frac{\theta_{\mathbf{k},\mathbf{k}'}}{2}\right),$$
$$E_{\mathbf{k}} = \hbar v_{\mathrm{F}}|\mathbf{k}|,$$
$$\omega_{\mathbf{q}} = v_{\mathrm{s}}|\mathbf{q}|,$$

and $f_{\mathbf{k}}$ is the drifting Fermi–Dirac distribution given by:

$$f_{\mathbf{k}} = \frac{1}{1 + \exp[\beta(E_{\mathbf{k}} - \hbar v_d k_x - \mu)]},$$

where we have chosen $v_d \parallel \hat{x}$. We choose to use the deformation potential $D = 19$ eV[3]. A is the sample area, and $\rho = 7.63 \times 10^{-8}$ g/cm^2 is the mass area density of graphene. The spin degeneracy g_{s} and valley degeneracy g_{v} are both 2. The chemical potential μ is related to the carrier density n by $\mu = \hbar v_{\mathrm{F}}\sqrt{\pi n}$, and the Fermi velocity $v_{\mathrm{F}} = 1000$ km/s. To simplify our computation, we consider only the longitudinal acoustic phonons, for which $v_{\mathrm{s}} = 21$ km/s $= 0.021 v_{\mathrm{F}}$. Phonon decay from other sources, such as the anharmonic interaction or point-defect scattering, are incorporated into $\tau_{\mathbf{q}}^{-1}$. The other two terms tend to be larger by at least an order of magnitude[2], so for simplicity we ignore the $\mathbf{q}$-dependence and choose $\tau_{\mathbf{q}}^{-1} = 100$ MHz. Plots of $\Gamma_{\mathbf{q}}^{\mathrm{amp}}$ for various drift velocity appear in Supplementary Figure 13a, and line cuts along $q_y = 0$ appear in Supplementary Figure 13b. $\Gamma_{\mathbf{q}}^{\mathrm{amp}}$ is positive for $v_d > v_{\mathrm{s}}$ in a cone along the direction of v_d. Above v_{s}, the strength of the effect increases linearly with v_d, as shown in Supplementary Figures 13 and 14b. Additionally, the strength of the effect scales linearly with the carrier density n, since the summation in the computation for $\Gamma_{\mathbf{q}}^{\mathrm{amp}}$ gives an overall factor of k_{F}^2. This is shown in Supplementary Figure 13c.

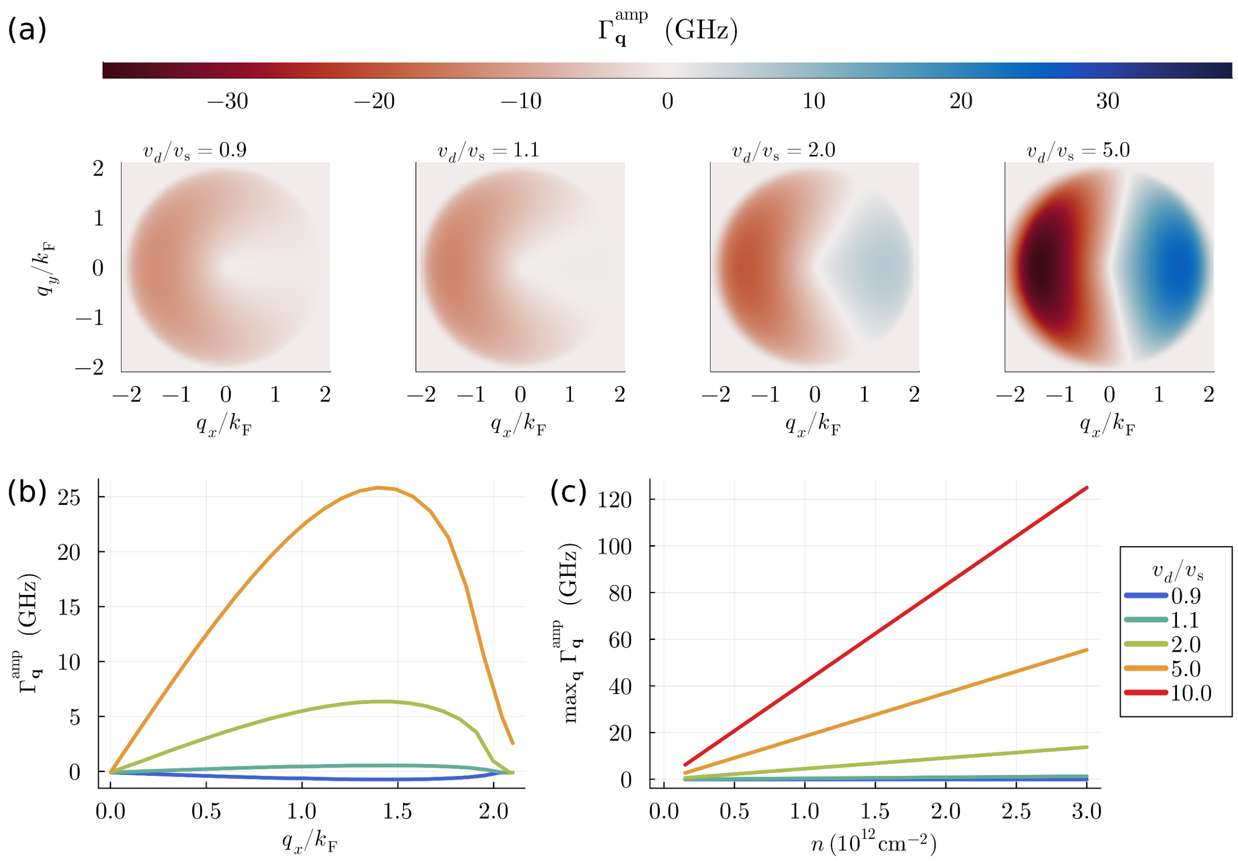

Supplementary Figure 13. Phonon amplification rates. A) The amplification rate $\Gamma_{\mathbf{q}}^{\mathrm{amp}}$ is plotted here for different values of v_d, with $\mathrm{T} = 2$ K, $n = 1.4 \times 10^{12}$ cm^{-2}. The effect from the electron-phonon interaction is nonzero out to $|\boldsymbol{q}| = 2k_F$, with this cutoff being smoothed out at nonzero temperature. Positive amplification rates appear in a cone in the direction of v_d once it exceeds v_s. The width of this cone and the magnitude of the effect increase as v_d increases. **B)** $\Gamma_{\mathbf{q}}^{\mathrm{amp}}$ line cuts along $q_y = 0$ for various drift velocities, $\mathrm{T} = 2$ K, $n = 1.4 \times 10^{12}$ cm^{-2}. For $v_d < v_s$, the amplification rate from electron-phonon coupling is always negative, and thus $\Gamma_{\mathbf{q}}^{\mathrm{amp}}$ never exceeds $-\tau_{\mathbf{q}}^{-1} = -100$ MHz. For $v_d > v_s$, electron-phonon coupling gives a positive amplification rate along the positive-q_x axis out to $q_x = 2k_F$. The overall scale of $\Gamma_{\mathbf{q}}^{\mathrm{amp}}$ increases as v_d increases; for $v_d > v_s$, the maximum value of $\Gamma_{\mathbf{q}}^{\mathrm{amp}}$ increases linearly with v_d (also seen in Supplementary Fig. 14b). **c)** The maximum value of $\Gamma_{\mathbf{q}}^{\mathrm{amp}}$ (maximized over $\mathbf{q}$) as a function of carrier density n for various drift velocities, $\mathrm{T} = 2$ K. At a given value of n, the maximum value of $\Gamma_{\mathbf{q}}^{\mathrm{amp}}$ is larger for larger v_d. For a given v_d, the maximum value of $\Gamma_{\mathbf{q}}^{\mathrm{amp}}$ scales linearly with n, since there is an overall factor of k_F^2 in the summation for $\gamma_{\mathbf{q}}^{\mathrm{em}}$ and $\gamma_{\mathbf{q}}^{\mathrm{abs}}$.

5.2 Phonon distribution

Using the amplification rate $\Gamma_{\mathbf{q}}^{\mathrm{amp}}$, we solve the Boltzmann transport equation for the phonons to obtain the x-dependent phonon population for each mode $\mathbf{q}$; for right-moving phonons this is:

$$n_{\mathbf{q}}(x) = n_{\mathbf{q},0} e^{\Gamma_{\mathbf{q}}^{\mathrm{amp}} x / v_{\mathbf{q}}} + \frac{1}{\Gamma_{\mathbf{q}}^{\mathrm{amp}} \tau_{\mathbf{q}}} \left(n_{\mathbf{q},0} + \tau_{\mathbf{q}} \gamma_{\mathbf{q}}^{\mathrm{em}}\right) \left(e^{\Gamma_{\mathbf{q}}^{\mathrm{amp}} x / v_{\mathbf{q}}} - 1\right),$$

and for left-moving phonons,

$$n_{\mathbf{q}}(x) = n_{\mathbf{q},0} e^{-\Gamma_{\mathbf{q}}^{\mathrm{amp}}(x-L)/v_{\mathbf{q}}} + \frac{1}{\Gamma_{\mathbf{q}}^{\mathrm{amp}} \tau_{\mathbf{q}}} \left(n_{\mathbf{q},0} + \tau_{\mathbf{q}} \gamma_{\mathbf{q}}^{\mathrm{em}}\right) \left(e^{-\Gamma_{\mathbf{q}}^{\mathrm{amp}}(x-L)/v_{\mathbf{q}}} - 1\right),$$

where $v_{\mathbf{q}} = v_{\mathrm{s}} \frac{q_x}{|\mathbf{q}|}$, L is the length of the sample, and $n_{\mathbf{q},0}$ is the equilibrium Bose-Einstein distribution.

5.3 Resistivity

Next, we compute the electric field E; to do this, we obtain a current-balance equation by multiplying both sides of the Boltzmann transport equation[4] for the electrons by v_x and summing over $\mathbf{k}$,

$$\frac{-eE}{\hbar} \sum_{\mathbf{k}} v_x \frac{\partial f_{\mathbf{k}}}{\partial k_x} = g_{\mathrm{s}} g_{\mathrm{v}} \sum_{\mathbf{k}} \sum_{\mathbf{k}'} v_x \left[F(\mathbf{k}, \mathbf{k}') - F(\mathbf{k}', \mathbf{k})\right],$$

where the scattering rate $F(\mathbf{k}, \mathbf{k}')$ is given by[3]

$$F(\mathbf{k}, \mathbf{k}') = f_{\mathbf{k}}(1 - f_{\mathbf{k}'}) W(\mathbf{k}, \mathbf{k}'),$$

$$W(\mathbf{k}, \mathbf{k}') = \frac{2\pi}{\hbar} \sum_{\mathbf{q}} \frac{D^2 \hbar |\mathbf{q}|}{2\rho A v_{\mathrm{s}}} \cos^2\left(\frac{\theta_{\mathbf{k},\mathbf{k}'}}{2}\right) \delta_{\mathbf{k},\mathbf{k}'+\mathbf{q}} \left[(n_{\mathbf{q}} + 1)\delta\left(E_{\mathbf{k}} - E_{\mathbf{k}'} - \hbar\omega_{\mathbf{q}}\right) + n_{-\mathbf{q}} \delta\left(E_{\mathbf{k}} - E_{\mathbf{k}'} + \hbar\omega_{\mathbf{q}}\right)\right].$$

The resistivity is then computed as E/j, where $j = nev_d$. A plot of the resistivity versus x for various v_d appears in Supplementary Figure 14a. We find that the resistivity is an exponential function of x, with the growth rate approximately corresponding to the maximum phonon amplification rate. For each v_d, we fit the logarithm of the resistance values versus x using a linear function to determine the growth rate. For $v_d > v_{\mathrm{s}}$, this growth rate closely matches $\max_{\mathbf{q}} \Gamma_{\mathbf{q}}^{\mathrm{amp}} / v_{\mathrm{s}}$, as shown in Supplementary Figure 14b.

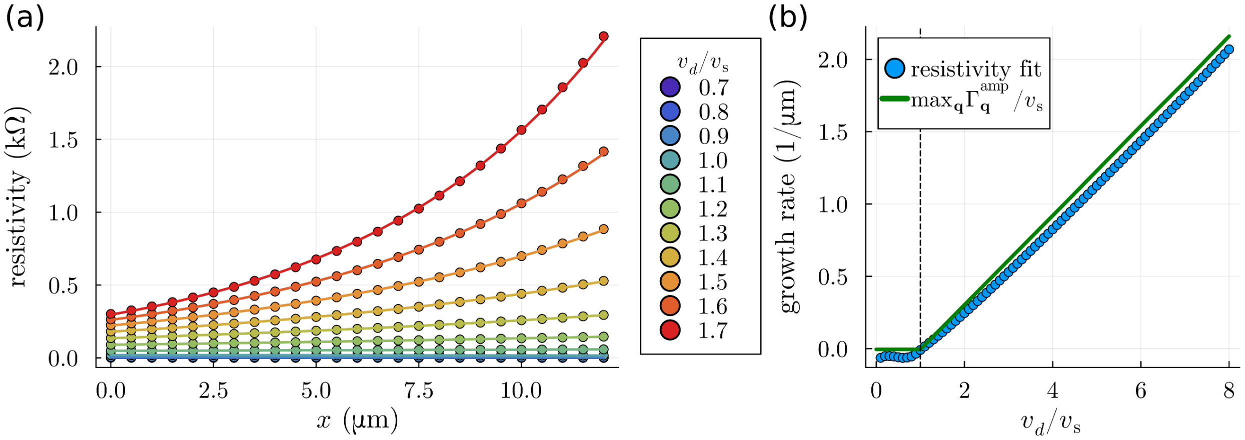

Supplementary Figure 14. Calculated resistivity growth due to phonon amplification. a) Resistivity versus x for various v_d, $n = 1.4 \times 10^{12}$ cm^{-2}, $T = 2$ K, with exponential fits. **b)** Resistivity growth rate (blue dots) as a function of v_d. The green line is the maximum Γ_q^{amp} divided by v_s for each v_d, which agrees well with the growth rates determined from fitting the computed resistivities. The dashed black line shows $v_d = v_s$.

Supplementary References:

1. Wallbank, J. R. *et al.* Excess resistivity in graphene superlattices caused by umklapp electron–electron scattering. *Nature Phys* **15**, 32–36 (2019).
2. Andersen, T. I. *et al.* Electron-phonon instability in graphene revealed by global and local noise probes. *Science* **364**, 154–157 (2019).
3. Hwang, E. H. & Das Sarma, S. Acoustic phonon scattering limited carrier mobility in two-dimensional extrinsic graphene. *Phys. Rev. B* **77**, 115449 (2008).
4. Zhao, C. X., Xu, W. & Peeters, F. M. Cerenkov emission of terahertz acoustic-phonons from graphene. *Appl. Phys. Lett.* **102**, 222101 (2013).
